# Don’t Sleep Well

**Published:** 1855-10

**Authors:** 


### "DON'T SLEEP WELL."

Since the *fullest* amount of sleep is as essential to the healthful working of mind and body as necessary food, it may be well to know how to secure it, as a general rule.

1. Clarify your conscience.

2. Take nothing later than two o'clock, P. M., except some bread and butter, and a small cup of weak tea of any kind, or half a glass of water, for supper.

3. Go to bed at some regular early hour. Get up the moment you wake of yourself, even if at midnight.

4. Do not sleep an instant in the day time.

Unless your body is in a condition to require special medical advice, nature will regulate your sleep to the wants of the system, in less than a month; and you will not only go to sleep at once, but will sleep soundly. "*Second naps*" and siestas make the mischief.

